# Knowledge of HIV/AIDS and predictors of uptake of HIV counseling and testing among undergraduate students of a privately owned university in Nigeria

**DOI:** 10.1186/1756-0500-7-639

**Published:** 2014-09-12

**Authors:** Olumide Abiodun, John Sotunsa, Franklin Ani, Ebunoluwa Jaiyesimi

**Affiliations:** Department of Community Medicine, Benjamin Carson (Snr) College of Medicine, Babcock University, Ilishan-Remo, Ogun State Nigeria; Department of Obstetrics and Gynecology, Benjamin Carson (Snr) College of Medicine, Babcock University, Ilishan-Remo, Ogun State Nigeria; Centre for Research in Reproductive Health, Sagamu, Ogun State Nigeria

**Keywords:** HIV/AIDS, HIV Counseling and testing, Predictors, Private university students, Ilishan

## Abstract

**Introduction:**

The spread of HIV/AIDS among the reproductive age group particularly young adults is a major public health concern in Nigeria. Lifestyles of students on university campuses put them at increased risk of contracting the HIV. The aim of this study was to assess the level of HIV/AIDS knowledge and to investigate the factors that were correlated with the uptake of and willingness to take up HIV counseling and testing.

**Methods:**

A cross-sectional study of 1,250 university students selected by 2-stage random sampling technique using self-administered questionnaire.

**Results:**

The participants consisted of 57.7% females and 42.3% males with ages ranging from 15 to 32 years and a mean of 19.13 ± 2.32 years. The awareness of HIV was universal. The knowledge about HIV/AIDS was very high with a mean score of 8.18 ± 1.60 out of 10; and 97.1% of participants having good knowledge of HIV/AIDS. The major source of HIV/AIDS information was the mass media. There was a significant difference in knowledge of HIV/AIDS by gender where male students had better knowledge about HIV/AIDS than females [t (1225) = 3.179, p = 0.002]. While 95% of the participants knew where to get an HIV test done, only 30.4% had tested for HIV within the six months preceding the study. However, 72.2% of them were willing to test for HIV. There was no significant association between demographic characteristics and having tested for HIV in the preceding six months but there was significant association between willingness to have an HIV test and the participants’ age groups, sex, marital status and their knowledge of HIV/AIDS. Participants who were aged 21 years and above and had good knowledge about HIV were more willing to take an HIV test. Females were more willing to take an HIV test than males.

**Conclusion:**

The participants’ knowledge about HIV /AIDS was quite good, the willingness to have HIV test done was high and the knowledge of a place where test can be done was nearly universal yet HIV testing was low. Innovative school based programs should be put in place to leverage on the willingness to test and translate it to periodic HIV testing.

## Background

The spread of HIV/AIDS among the productive age group particularly young adults is a major public health concern in Nigeria. Sub-Saharan Africa which accounts for just a little above 10% of the world’s population is however, the worst hit region in the world. The region has a rapidly increasing rate of infection among adolescents [1]. The ages between 15 and 24 years represent the highest at risk group for the infection of the virus [2]. They account for over 40% of all new HIV infections among adults [3–5]. Researches in Nigeria and neighboring Ghana indicate that the lifestyles of students on university campuses put them at increased risk of contracting the HIV [6–8]. This is because the university environment has been shown to promote sexual activity among the general student population [9, 10]. Hence, it is understandable that the predominant mode of transmission of HIV in sub-Saharan Africa, accounting for approximately 90% of all infections is by the heterosexual means [1].

Peer pressure within campus environments lead many young women and indeed men to engage in transactional sex [11]. An abundance of evidence exists to show that although knowledge about a disease is a prerequisite for but is not a predictor of behavioural change [12, 13]. Recent studies on HIV among young adults in Nigeria has predominantly focused on public universities [9–11]. There is a paucity of data on private university students, despite the presence of more than 50 private universities in Nigeria as indicated by the National Universities Commission a parastatal under the Federal Ministry of Education that facilitates the establishment of both public and private Universities [14].

National data show that universal awareness is yet to be achieved (93%) and that comprehensive knowledge is abysmally poor at 25% [15]. The same report indicated that only 24.2% of males and females aged 15–24 were able to correctly identify factual information and reject common misconceptions [15]. This reflected a slight increase from 22.5% in 2005 [16]. This has significant implications for the formulation of policy and HIV prevention interventions. Schools especially universities are the most likely arena for initiating sexual contacts and also represent an arena for sexual risk taking and thus university students are at risk of HIV [17, 18].

Access to HIV counseling and testing (HCT) is a major pillar of the National strategic framework adopted by Nigeria for HIV control. The federal government of Nigeria has introduced and implemented various programmes to increase the uptake of HCT. Notwithstanding these interventions, HIV testing uptake is still low (11% of women and men aged 15 to 49 years) and unknown to many Nigerians [15, 16], due to the fear of the testing outcome and HIV related stigma [16]. There is a paucity of information on HIV counseling and testing among university students and the factors that influence them to test for HIV in the sub-Saharan region. It is also not clear if the factors that influence the general population and public university students to test for HIV [19, 20] can be attributed to HIV testing uptake among private university students.

Despite young adults’ increased vulnerability to HIV infection as a consequence of risky sexual behaviours, there are no studies that have examined HIV/AIDS knowledge and the uptake of HIV testing among private university students in Nigeria.

### Aim of the Study

The aim of this study was to assess the level of HIV/AIDS knowledge and to investigate the factors that were correlated with the uptake of HIV counseling and testing among private university students in Ilishan, Nigeria. The findings of the study should form a basis for the formulation of policy on increasing uptake of HIV counseling and testing services to university students in Nigeria. Especially because university students belong to an age-group that is at greater risk for HIV infection.

## Methods

### Study setting and sample

A cross-sectional study was conducted at Babcock University, Ilishan Nigeria between September 2013 and January 2014. Babcock University is one of the 51 private universities in Nigeria. The University has total of 12000 regular undergraduate students.

### Sample size and sampling procedure

The sample size was determined taking 24.2% expected prevalence of comprehensive knowledge as reported for young people in Nigeria [15], assuming 5% margin of error and 95% confidence level, design effect of 2 and 10% for non-response rate. The minimum calculated sample size was 605. However, 1,250 students were targeted to increase the reliability of the data collected. The University is almost fully residential with 15 student hostel in the two campuses (12 in Ilishan and 3 in Iperu). Five hostels were selected by simple random technique via balloting (one in Iperu and four in Ilishan). Proportional allocation based on the population of the selected hostels was used to decide the required sample size for each hostel. The students were then selected from each hostel using stratified sampling from a list of students in the hostels. Questionnaire administration was done during the monthly fellowship meetings which is compulsory for all students.

### Measurement

A self-administered AIDS Knowledge and Attitude Questionnaire that had been validated and used in Ghana [8] was adapted for the study. Three Reproductive Health Specialists were requested to vet the instrument and establish content validity. The Specialists made various suggestions which were taken into account, and necessary corrections were made. There was consensus among the Specialists on the suitability of the instrument for use in the study context. Test-retest reliability was conducted to assess the questionnaire stability estimating the intra-class correlation coefficient (ICC). Fifty participants completed the questionnaire twice in two-week intervals. ICC values of 0.40 or above were considered satisfactory. The questionnaire was then pretested among 125 students at a Public University in Nigeria. Necessary adjustments were made. Questions were asked to assess respondents' knowledge about HIV/AIDS. The data were collected on the dependent variable i.e. Uptake of HCT and the independent variables including socio demographic variables, knowledge related to HIV/AIDS transmission and prevention. The respondents' knowledge of HIV/AIDS was assessed by assigning a score of 1 to each correct answer of 10 yes/no/don’t know questions. Scores 0 to 3 were regarded as very poor; 4 as poor; 5 to 6 as good and 7 to 10 as very good knowledge. The means (M) and standard deviation (SD) were derived.

### Statistical analysis

The data were coded, edited and entered, cleaned and analyzed using IBM SPSS Statistics version 20. An independent sample *t*-test and Analysis of variance (ANOVA) was used to compare the differences in mean of the knowledge on HIV/AIDS according to the various demographic characteristics. Chi-square (***χ***^**2**^) was used to explore the factors associated with having taken an HIV test and the desire to be tested in the future as well as the sources of HIV/AIDS information. To assess the relative contribution of each of these predictor variables, a logistic regression analysis was carried out. Variables used for bivariate analyses were included in the logistic model and categorical variables were re-coded into a binary form. These variables were entered into the model using a block entry approach. Odds ratios (OR) and 95% confidence intervals (CI) were produced for each predictor variable. P-values <0.05 was considered statistically significant.

### Ethical issues

Ethical clearance and permission was obtained from the Babcock University Teaching Hospital Ethical Committee. Informed consent was obtained from each participant. Prior to the administration of questionnaire, the purpose of the study was explained to the participants. The students were free to decline participation in the study. Confidentiality was maintained by omitting their personally identifiable information such as names from the questionnaire.

## Result

### Characteristics of study participants

The study recruited 1,250 undergraduate students out of which 1,225 properly filled questionnaires were returned giving a response rate of 98.0%. Table 1 shows the characteristics of the study participants. The participants consisted of 57.7% females and 42.3% males with ages ranging from 15 to 32 years and a mean of 19.13 years and standard deviation of 2.32 years. An overwhelming majority of the participants (99.5%) were single and never married while 93.6% were Christians.

**Table 1:** **Socio-demographic information of the participants (n = 1225)**

Variables	Frequency	Percentage
Age in years		
15-17	286	23.3
18 to 20	689	56.2
21 to 23	187	15.3
> 23	63	5.1
Gender		
Male	518	42.3
Female	707	57.7
Marital status		
Single	1219	99.5
Married	6	0.5
Religion		
Christianity	1146	93.6
Islam	70	5.7
Others	9	0.7
Source of information about HIV/AIDS		
Mass media	903	73.7
Health workers	269	22.0
Others	53	4.3

### HIV transmission and prevention knowledge

The awareness of HIV/AIDS was universal. All the respondents knew a place where HIV test could be done. The mass media followed by health workers were the main source of awareness and information about HIV/AIDS. The other sources were friends and family. The majority (93.7%) of the participants correctly identified unprotected sexual intercourse with infected persons as a means of transmission, 96.1% identified transfusion with infected blood and 70.4% identified mother to child transmission.

The knowledge of method of HIV prevention was quite high. The participants correctly identified abstinence (96.2%), being faithful to an uninfected partner (85.7%) and consistent condom usage (88.4%) as ways of preventing HIV infection. However, the knowledge of treatment for HIV/AIDS was not as high as only 60.5% of the participants indicated that they know that there was no cure for AIDS, and 82% indicated that one may not always know by appearance if someone is infected with HIV.

### Relationship between HIV knowledge and socio-demographic characteristics

The participants had an excellent knowledge about HIV/AIDS. Although the total HIV knowledge score ranged from 0 to 10, the mean was 8.18 with standard deviation of 1.60. When the knowledge scores were grouped into good scores (5-10) and poor scores (0–4), only 35 (2.9%) had poor knowledge scores while 97.1% had good knowledge. Table 2 shows the comparison between mean knowledge score on HIV/AIDS and various socio-demographic characteristics using the independent t-test and Analysis of variance (ANOVA).

**Table 2:** **Differences in HIV/AIDS knowledge by demographic characteristics (n = 1225)**

Variable	Mean (SD)	Test values	p-value
Age in years			
15 to 17	7.95 (1.712)	2.832^a^	0.037
18 to 20	8.28 (1.53)		
21 to 23	8.17 (1.64)		
> 23	8.25 (1.64)		
Sex			
Male	8.35 (1.60)	3.179^b^	0.002
Female	8.06 (1.60)		
Religion			
Christianity	8.21 (1.59)	2.607^a^	0.074
Islam	7.89 (1.58)		
Others	7.33 (2.92)		
Source of information about HIV/AIDS			
Mass media	8.15 (1.63)	1.294^a^	0.274
Health workers	8.21 (1.57)		
Others	8.51 (1.31)		
TOTAL	8.18 (1.60)		

^a^F-value from one-way ANOVA ^b^independent t-test values.

The results of an independent sample t-test which compared the differences in the mean knowledge on HIV/AIDS according to sex and marital status showed that the male participants (M = 8.36, SD = 1.60) had better knowledge about HIV/AIDS than their females counterpart (M = 8.06, SD = 1.60) [t (1225) = 3.179, p = 0.002].

The One-Way Analysis of variance (ANOVA) indicated that there was a statistically significant difference in the HIV/AIDS Knowledge scores according to age group (p = 0.037), but insignificant difference according to religion (p = 0.074) and source of knowledge on HIV/AIDS (p = 0.287).

### Counseling and testing for HIV

While 1,164 (95%) of the participants knew where to get an HIV test done, only 373 (30.4%) had tested for HIV within the six months preceding the study. Over seventy percent (72.2%) of them stated that they were willing to test for HIV.

Table 3 shows the relationship of HIV testing and desire to have test done with the various socio-demographic variables. All of the socio-demographic characteristics had no statistically significant relationship with having tested for HIV in the six month period preceding the study (p > 0.05). However, there was statistically significant relationship (p < 0.05) between willingness to have an HIV test and the participants’ age groups, sex and their knowledge of HIV/AIDS. There was no statistically significant relationship (p > 0.05) between willingness to have an HIV test and the participants’ religion and source of information about HIV/AIDS.

**Table 3:** **Relationship of socio-demographic characteristics with HIV testing and intention to test for HIV**

Variable	Have you had HIV testing in the last six months? (N = 1225)			Are you willing to have HIV test in the near future? (N = 1225)		
	Yes	No	***X*** ^2^	Yes	No	***X*** ^2^
	N (%)	N (%)	(p-values)	N (%)	N (%)	(p-values)
Age in years						
15 to 17	94 (32.9)	192 (67.1)	3.105 (0.376)	191 (66.8)	95 (33.2)	8.304 (0.040)
18 to 20	196 (28.4)	493 (71.6)		505 (73.3)	184 (26.7)	
21 to 23	61 (32.6)	126 (67.4)		146 (78.1)	41 (21.9)	
> 23	22 (34.9)	41 (65.1)		43 (68.3)	20 (31.7)	
Gender						
Male	166 (32.0)	352 (68.0)	1.081 (0.298)	344 (66.4)	174 (33.6)	15.243 (0.000)
Female	207 (29.3)	500 (70.7)		541 (76.5)	166 (23.5)	
Religion						
Christianity	345 (30.1)	801 (69.9)	1.383 (0.501)	826 (72.1)	320 (27.9)	4.882 (0.087)
Islam	24 (34.3)	46 (65.7)		55 (78.6)	15 (21.4)	
Others	4 (44.4)	5 (55.6)		4 (44.4)	4 (55.6)	
Source of information about HIV/AIDS						
Mass media	278 (30.8)	625 (69.2)	0.926 (0.629)	653 (72.3)	250 (27.7)	0.900 (0.638)
Health workers	82 (30.5)	187 (69.5)		191 (71.0)	78 (29.0)	
Others	13 (24.5)	40 (75.5)		41 (77.4)	12 (22.6)	
Knowledge of HIV						
Good	363 (30.5)	827 (69.5)	0.060 (0.807)	873 (73.4)	317 (26.6)	25.891 (0.000)
Poor	10 (28.6)	25 (71.4)		12 (34.3)	23 (65.7)	

The age distribution showed that the proportion of those who were willing to have an HIV test was highest among students aged 21–25 (78.8%) and reduced to 58.8% among those aged 15 or less. It was however lowest among those older than 25 years (50.0%). The willingness to have HIV testing among the participants therefore increased with age (except for those older than 25 years). The older the participant (until age 25), the more the likelihood to be willing to test for HIV (p = 0.007).

A higher proportion of females (76.5%) than males (66.4%) were willing to have HIV test done (p = 0.000). Table 3 also shows that the knowledge about HIV/AIDS was related to the willingness of participants to have HIV screening (p = 0.000). A higher proportion of participants with good knowledge (73.4%) were willing to have an HIV test than those with poor knowledge (34.3%).

Table 4 shows binary logistic regression of predictors for having taken an HIV test in the preceding six months and willingness to test for HIV. The Table shows that none of the socio-demographic characteristics considered was associated with the odds of having had an HIV test in the preceding six months. However, ages from 21 years and above, the female gender and good knowledge about HIV were found to be predictors of willingness to have an HIV test done. Participants who were less than 21 years old were slightly less likely (OR = 0.671; 95% CI = 0.478 – 0.941, p = 0.021) to be willing to take an HIV test than those who were 21 years and older. Female participants were almost two times more likely (OR = 1.650; 95% CI = 1.275 – 2.136, p = 0.000) to be willing to take an HIV test than their male counterparts. Participants with good knowledge about HIV were five times more likely (OR = 5.278; 95% CI = 2.663 – 11.349, p = 0.000) to be willing to take an HIV test than those with poor knowledge.

**Table 4:** **Logistic regression of the predictors of having tested for HIV and willing to test for HIV**

Variables	Coefficient (β)	SE	p-value	Odds Ratio (OR)	95% CI
HIV testing in the last six months					
Age (less than 21 years )	-0.205	0.153	0.180	0.815	0.604- 1.099
Gender (female)	-0.143	0.126	0.257	0.867	0.677- 1.110
Religion (Christianity)	-0.299	0.246	0.224	0.741	0.458- 1.201
Source of HIV information (Media)	0.074	0.143	0.604	1.077	0.814- 1.424
Knowledge of HIV	0.103	0.381	0.788	1.108	0.525- 2.340
Willingness to HIV test					
Age (less than 21 years )	-0.400	0.173	0.021	0.671	0.478- 0.941
Gender (female)	0.501	0.132	0.000	1.650	1.275- 2.136
Religion (Christianity)	-0.226	0.285	0.429	0.798	0.456- 1.396
Source of HIV information (Media)	-0.033	0.149	0.826	0.977	0.722- 1.296
Knowledge of HIV	1.704	0.370	0.000	5.278	2.663- 11.349

## Discussion

The study participants were universally aware of HIV/AIDS. Their knowledge about the cause, mode of transmission and prevention of HIV was quite high, with an average score of 8.18 out of a total of 10.0 and standard deviation of 1.60. However, the uptake of HIV screening in the preceding six months to the study was only 30.4%. The study also demonstrated a significant gender difference in HIV knowledge among private university students in Nigeria, with males being more knowledgeable than females, though females were more willing to have an HIV test than males. The willingness to have HIV test was also associated with increasing age and having good knowledge of HIV. Good knowledge of HIV, the female gender and ages from 21 years and above were found to be associated with an increased odd to be willing to have an HIV test.

The universal awareness and very high level of HIV knowledge among the private university students as shown in this study is attributable to sustained health education programmes. In the last 10 years, the Nigerian government through the National Agency for the Control of HIV/AIDS (NACA) and its development partners increased public awareness on the causes and prevention of STIs including HIV/AIDS. Higher levels of knowledge have been reported in other university students in Nigeria [21]. Besides, studies have associated higher levels of education with higher levels of HIV knowledge [22, 23]. In Ghana, similar efforts to those of NACA have been complemented by the establishment of HIV/AIDS Clubs in tertiary institutions. This can be adopted and be scaled up to cover secondary schools as well [24]. The aim of such Clubs would be to train university students to contribute to HIV/AIDS awareness and knowledge in their respective campuses as peer educators [24, 25].

The level of HIV knowledge differed between male and female university students in this study. This finding is consistent with studies conducted among university studies in other parts of Nigeria [21, 26], Ghana [24, 27] and other parts of the world [22, 23] which found that AIDS knowledge differs on the basis of gender among university students. However, other studies have indicated no gender differences on HIV knowledge and attitudes among university students [28, 29]. This gender difference found in this study which favors males is however, contrary to other studies which showed that females had a better knowledge about HIV/AIDS than male university students [21, 24, 26, 27]. It is thought that female university students show more concern about their health than males, thus seeking to know more about issues related to health including HIV/AIDS [24]. It is suggested that the higher knowledge of HIV shown by males could be due to difference in access to information, media and participation in different anti-HIV/AIDs club [22].

Majority of the private university students reported having received AIDS information from the mass media followed by health workers. Only a few of them received such information from their schools and parents. This is in spite of the fact that they spend a considerable part of the time in school or with their parents at home. This is however a finding that is consistent with other studies which have shown that university students rely on the mass media as the major source of information on HIV/AIDS [26, 30, 31]. However, the mass media did not prove to be more effective at disseminating correct information on HIV/AIDS than other means. This is in spite of the fact that the mass media has proved to be a more effective tool for addressing specific HIV issues including knowledge, self-efficacy of condoms use, social norms, interpersonal communication and awareness of health services [24, 32]. The mass media therefore have an important role to play in dissemination of information about HIV/AIDS among private university students. The various media including television, radio and social media should therefore be intensely engaged to spread the knowledge about HIV and other health reproductive issues. The Internet is also an important source because young people are very active Internet users [33]. The finding that schools and parents were not the main sources of knowledge about HIV/AIDS are similar to other studies [34]. This shows that schools based reproductive health education programs are not quite emphasized. Although talking about HIV/AIDS with parents improves students’ knowledge about HIV/AIDS [35], Parents have difficulty in speaking openly about HIV/AIDS with their children [33]. Such discussions are rare because of lack of relevant information by the older generation, family structure, the conservative culture, religious and traditional beliefs on issues of sexuality, condom usage and marriage [24, 36]. There is a need for parents to discuss reproductive sexual issues with their children and young adults and for schools to devise means to promote school based reproductive health education. This is important because protection by adults has been shown to decrease their young people’s risk of HIV infection [37].

The very good knowledge about HIV and near universal knowledge of where to have an HIV test done did not translate to HIV test uptake. All Babcock university students are required to have an HCT as part of pre-admission health screening exercise. The rate is relatively high when compared with university students in Bayelsa and Lagos in Nigeria and Ghana where 53.8%, 26.1 and 45% of the students respectively reported having ever tested for HIV [24, 38, 39]. In these Universities, HCT is not included in the pre-admission health screening process. In the six months preceding this study, 30.4% of the students had taken a test. The gap between knowledge and uptake may be attributable to fear of stigmatization as well as discrimination associated with the counseling and testing and AIDS [24]. Fear of stigma have been shown to influence young adults to become less likely to engage in preventive behaviours [2] and an increase in knowledge about HIV does not predict behavioural change [12, 13]. The gap may also be attributable to the fact that the majority of the students report no sexual activity and have a perception that they are not at risk of HIV/AIDS, hence they are unable to appreciate the need for a periodic screening. The fact that over 70% of the students were willing to take an HIV test is quite encouraging. The willingness to have HIV test done was predicted by age from 21 years and above, female gender, being single and having good knowledge. It is therefore imperative to explore all strategies to sustain and further enhance the knowledge about HIV. It has been shown that sensitization program on benefits of HCT will improve uptake of HCT among the young people in Nigeria [40]. Hence, proactive measures should be pursued to ensure periodic screening by university students. Knowing where to test for HIV it does not necessarily influence their desire to get tested in the future [24]. Therefore, beyond making HCT facilities available on various university campuses, the operators of such facilities must go beyond the wait for students to have voluntary counseling and testing (VCT) and take the screening exercises to the students through various innovative campaigns.

### Limitations of the study

Certain shortfalls of this study should be put into consideration when interpreting the findings. The study was cross–sectional in design hence, causality cannot be established. The use of self-reported data means that the accuracy of the data cannot be ascertained especially when the sensitive nature of the subject is considered. This is further underlined by the fact that the study was conducted in a Faith based university where Christian values are an integral part of learning. The study was conducted in only one private university. This will limit the generalizability of the findings to the entire population of private university students in Nigeria. Besides, the pre-admission HCT program of the institution (which is not so common) may have had some influences on the participants, uptake of HCT within six months preceding the study. Despite these shortcomings, this study fills a gap in research in the area of HIV/AIDS knowledge among private university students, and the influence of demographic variables associated with HIV counseling and testing. Probability sampling was used and the sample size was considerable.

## Conclusion

The university administration should explore all strategies to sustain and further enhance the knowledge about HIV through innovative school based programs. The willingness of the students to take HIV test should be leveraged upon to promote periodic screening. The operators of HCT facilities must go beyond the wait for students to have VCT and take the screening exercises to the students through various innovative campaigns. Future studies in institutions with similar pre-admission HCT programs may be necessary underline the effect of such programs on subsequent uptake of HCT and intention to have HCT. Future qualitative studies are also required to explore the barriers to uptake of HCT among private university students.
